# Supplementary figures and images for: Geldanamycin treatment does not result in anti-cancer activity in a preclinical model of orthotopic mesothelioma
Source: PLoS One. 2023 May 5;18(5):e0274364. doi: 10.1371/journal.pone.0274364 (PMC10162533; doi:10.1371/journal.pone.0274364)

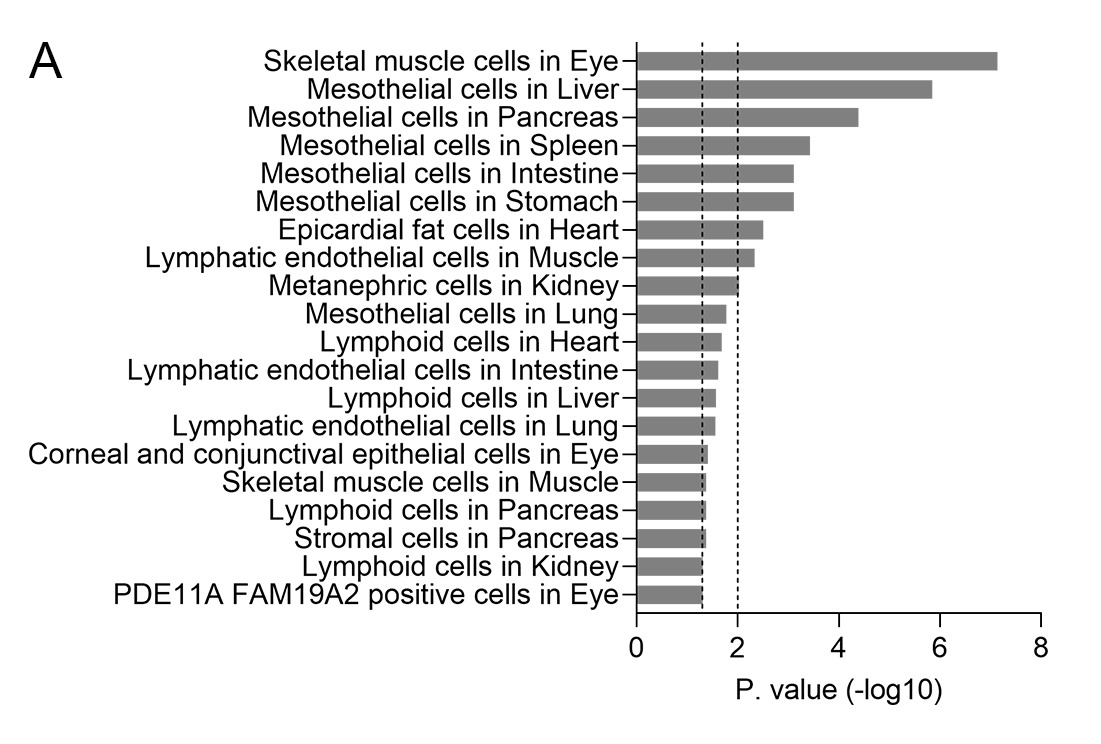

Supplement: S1 Fig — (A) Enrichment analysis of IPL (log fold change >1) differentially expressed genes using the Descartes dataset in Enrichr, p values 0.05 and 0.01 are represented with dotted line on–log10 = 1.3 and 2, respectively. (TIF) [file pone.0274364.s001.tif]

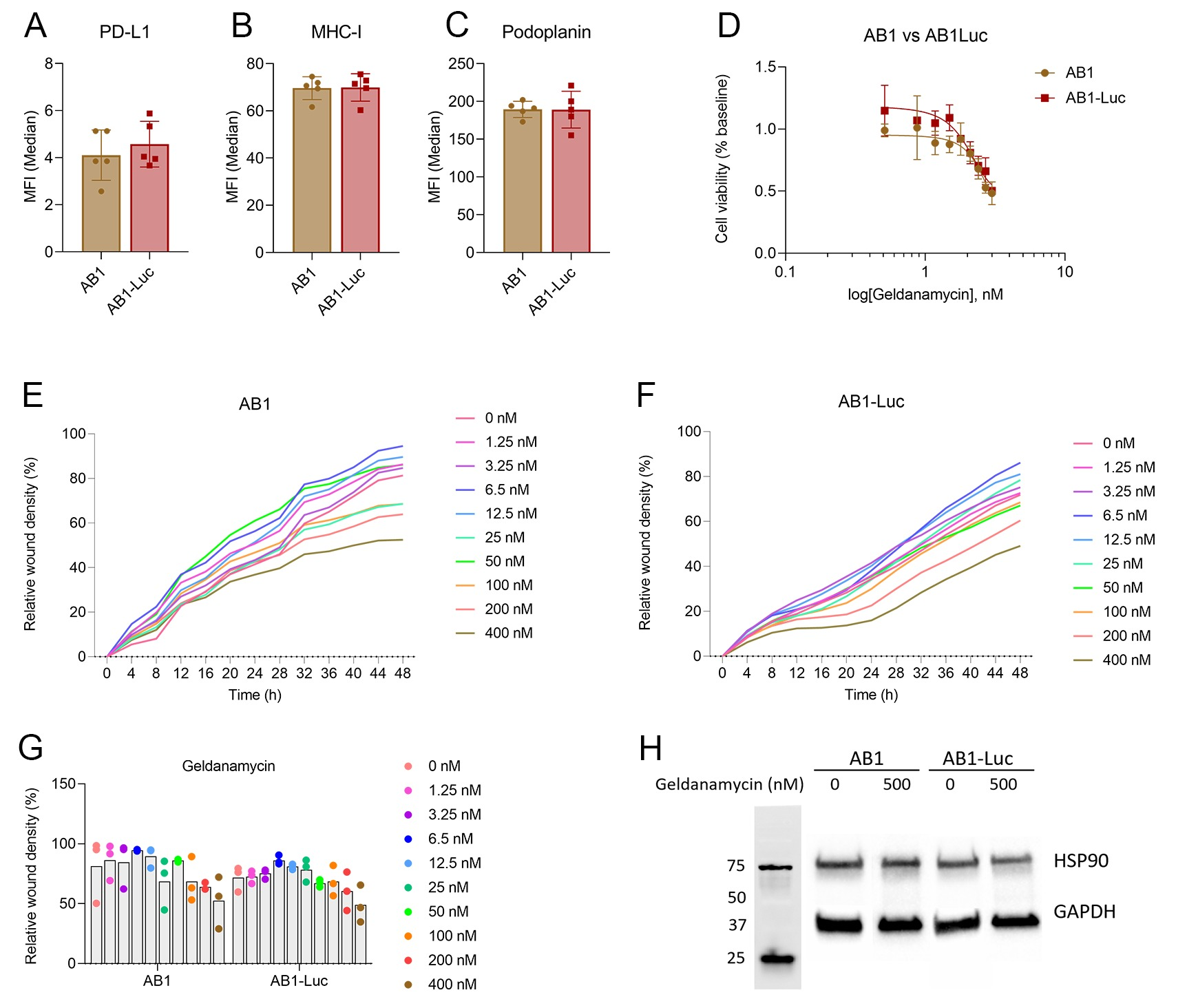

Supplement: S2 Fig — (A–C) Flow cytometry for surface markers (A) PD-L1, (B) MHC-I, and (C) Podoplanin in AB1 and AB1-Luc cell lines. Data is represented as MFI (Median). N = 5. (D) MTT assay in AB1 and AB1-Luc cell lines. The curve is presented as a non-linear regression; log(geldanamycin) versus response. IC50 values are in nM. Data are presented as means ± SD of 3 replicates. (E–G) Scratch assay for migration in (E) AB1 and (F) AB1-Luc cell lines. Geldanamycin was serial diluted from 400 nM to 1.25 nM, with 0 nM as control. (G) AB1 and AB1-Luc side by side comparison. (H) Western blot for cell lines AB1 and AB1-Luc shows housekeeping protein GAPDH at 37 kDa, and HSP90 protein at 90 kDa. (TIF) [file pone.0274364.s002.tif]

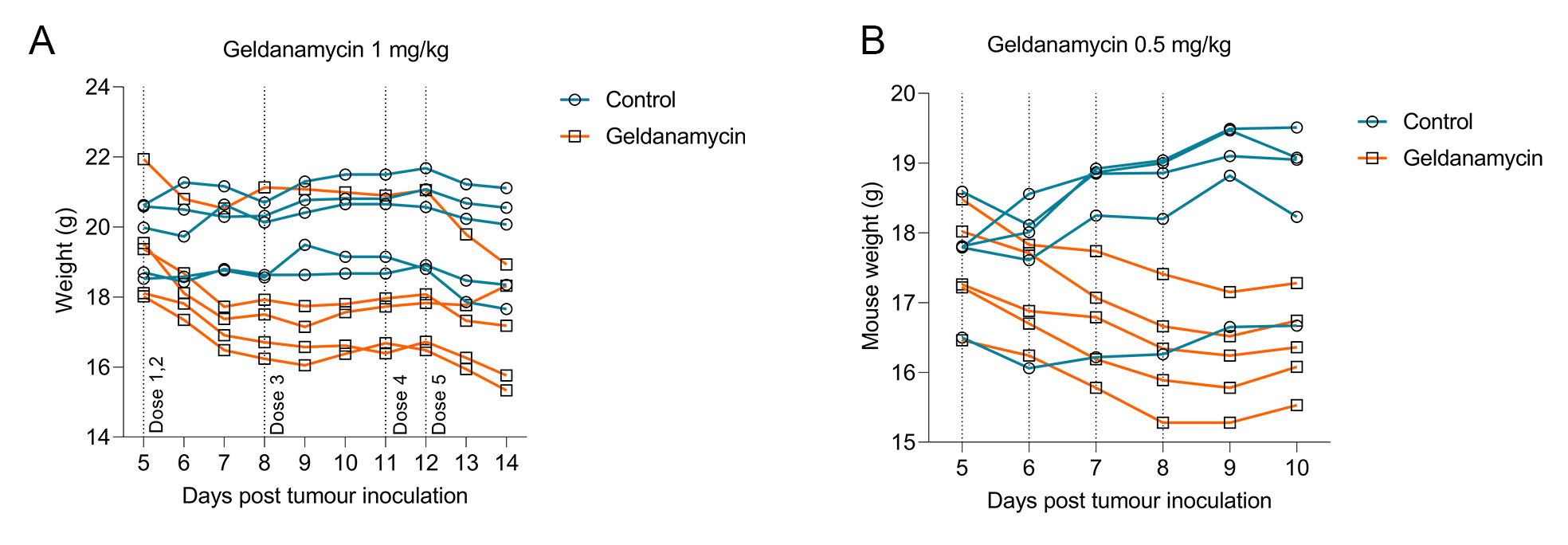

Supplement: S3 Fig — (A) Mice weight monitored daily during treatment at 1 mg/kg, each dotted line indicates the number of doses. (B) Mice weight monitored daily during treatment at 0.5 mg/kg, each doted like indicates two doses on that specific day. (TIF) [file pone.0274364.s003.tif]
