# Supplementary material for: Geldanamycin treatment does not result in anti-cancer activity in a preclinical model of orthotopic mesothelioma
Source: PLoS One. 2023 May 5;18(5):e0274364. doi: 10.1371/journal.pone.0274364 (PMC10162533; doi:10.1371/journal.pone.0274364)
Supplement: S1 Table — (DOCX) [file pone.0274364.s004.docx]

**S1 Table. Mice clinical signs scoring criteria**

| **Score** |  | **0** | **1** | **2** |
| --- | --- | --- | --- | --- |
| **Appearance** | *Weight Loss* | Steady weight | >10% weight loss | 15%-19.99% weight loss |
|  | *Coat* | Normal | Mild ruffled coat | Moderate ruffled coat, ungroomed |
|  | *Body Condition* | Normal | Thin | Loss of body fat, failure to grow |
|  | *Body Posture* | Normal | Hunched | Hunched and still |
|  | *Movement* | Normal | Reduced/slow | Reluctant to move when touched |
| **Activity** | *Proximity to Others* | Normal | Somewhat separate | Completely separate |
| **Other** | *Injection Site* | Normal | Some redness at margins | Redness and |
