# Supplementary material for: Geldanamycin treatment does not result in anti-cancer activity in a preclinical model of orthotopic mesothelioma
Source: PLoS One. 2023 May 5;18(5):e0274364. doi: 10.1371/journal.pone.0274364 (PMC10162533; doi:10.1371/journal.pone.0274364)
Supplement: S2 Table — (DOCX) [file pone.0274364.s005.docx]

**S2 Table. Mice intervention criteria**

| **Score** | **Assessment** | **Intervention** |
| --- | --- | --- |
| **0** | Animal normal | No intervention needed. Standard monitoring procedure. |
| **1** | Animal slightly deviated from normal | Increase monitoring to 3x weekly. The animal will be monitored again within 24 hours if a score above zero is recorded. |
| **2** | Animal demonstrates mild deviation from normal | Increase monitoring to daily until animal returns to normal |
| **3-4** | Animal demonstrates moderate deviation from normal | Monitor 2x daily, weigh 3x a week. Contact Bioresources staff for advice or administer pain relief (Buprenorphine). Consider euthanasia. |
| **>4** | Animal demonstrates significant deviation from normal or is noticeably distressed or unwell | Euthanasia |
