# Supplementary material for: Geldanamycin treatment does not result in anti-cancer activity in a preclinical model of orthotopic mesothelioma
Source: PLoS One. 2023 May 5;18(5):e0274364. doi: 10.1371/journal.pone.0274364 (PMC10162533; doi:10.1371/journal.pone.0274364)
Supplement: S3 Table — (DOCX) [file pone.0274364.s006.docx]

**S3 Table. Flow cytometry panel antibody information**

| **Fluorochrome** | **Antigen** | **Dilution** | **Clone** | **Company** | **Catalogue number** | **Cocktail** |
| --- | --- | --- | --- | --- | --- | --- |
| - | CD16/CD32 (Fc block) | 1:100 | 93 | eBioscience^TM^ | 14-0161-82 | Blocking |
| FVS780 | Live/Dead | 1:1000 | - | BD Horizon™ | 565388 | Viability |
| BUV395 | PD-L1 | 1:200 | MIH5 | BD Horizon™ | 745616 | Surface |
| BV421 | Podoplanin | 1:200 | 8.1.1 | BioLegend | 127423 |  |
| PE | MHC-I | 1:200 | H-2Kd/H-2Dd | eBioscience^TM^ | 12-5998-82 |  |
